# Supplementary material for: The influence of resource use on yield versus sale price trade-off in Australian vineyards
Source: PLoS One. 2025 Jun 5;20(6):e0323500. doi: 10.1371/journal.pone.0323500 (PMC12140259; doi:10.1371/journal.pone.0323500)
Supplement: Table S1 — P-values for the Pearson correlation coefficients of the non-transformed water used variable with other variables. This table highlights the statistical significance of correlations in the dataset. (PDF) [file pone.0323500.s001.pdf]

Variable	Water Used
Yield	7.538E-01
Area	6.981E-01
Scope One Emissions	8.883E-01
$\frac{\text{Yield}}{\text{Area}}$	6.836E-01
Average Price Per Tonne	5.600E-02
$\frac{\text{Average Price Per Tonne}}{\text{Area}}$	1.522E-01
