# Supplementary figures and images for: The influence of resource use on yield versus sale price trade-off in Australian vineyards
Source: PLoS One. 2025 Jun 5;20(6):e0323500. doi: 10.1371/journal.pone.0323500 (PMC12140259; doi:10.1371/journal.pone.0323500)

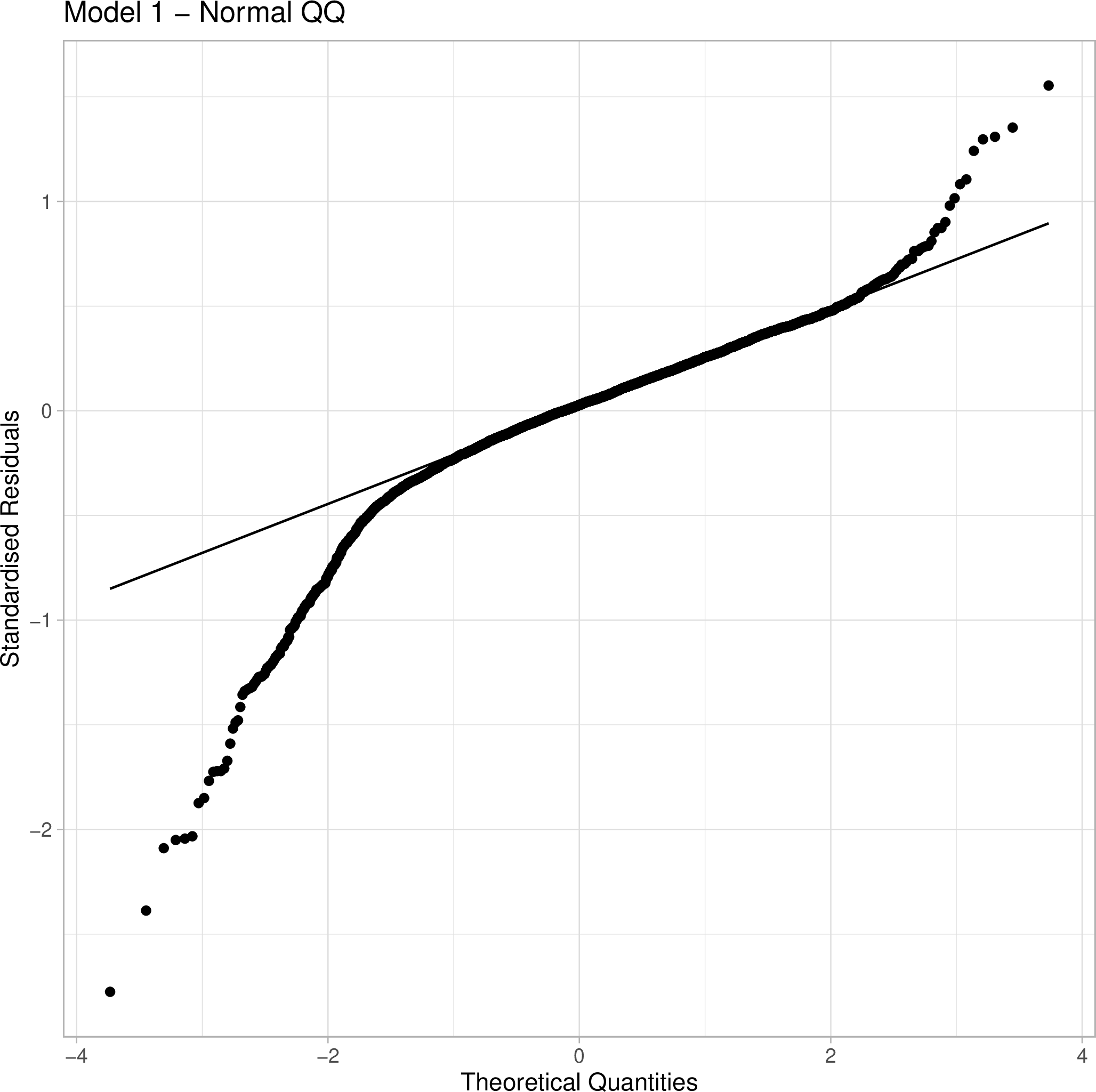

Supplement: Fig S1 — Quantile-Quantile (QQ) plot of Model 1 residuals, showing how closely the residuals align with a normal distribution. Deviations from the line indicate departures from normality. (TIFF) [file pone.0323500.s002.tif]

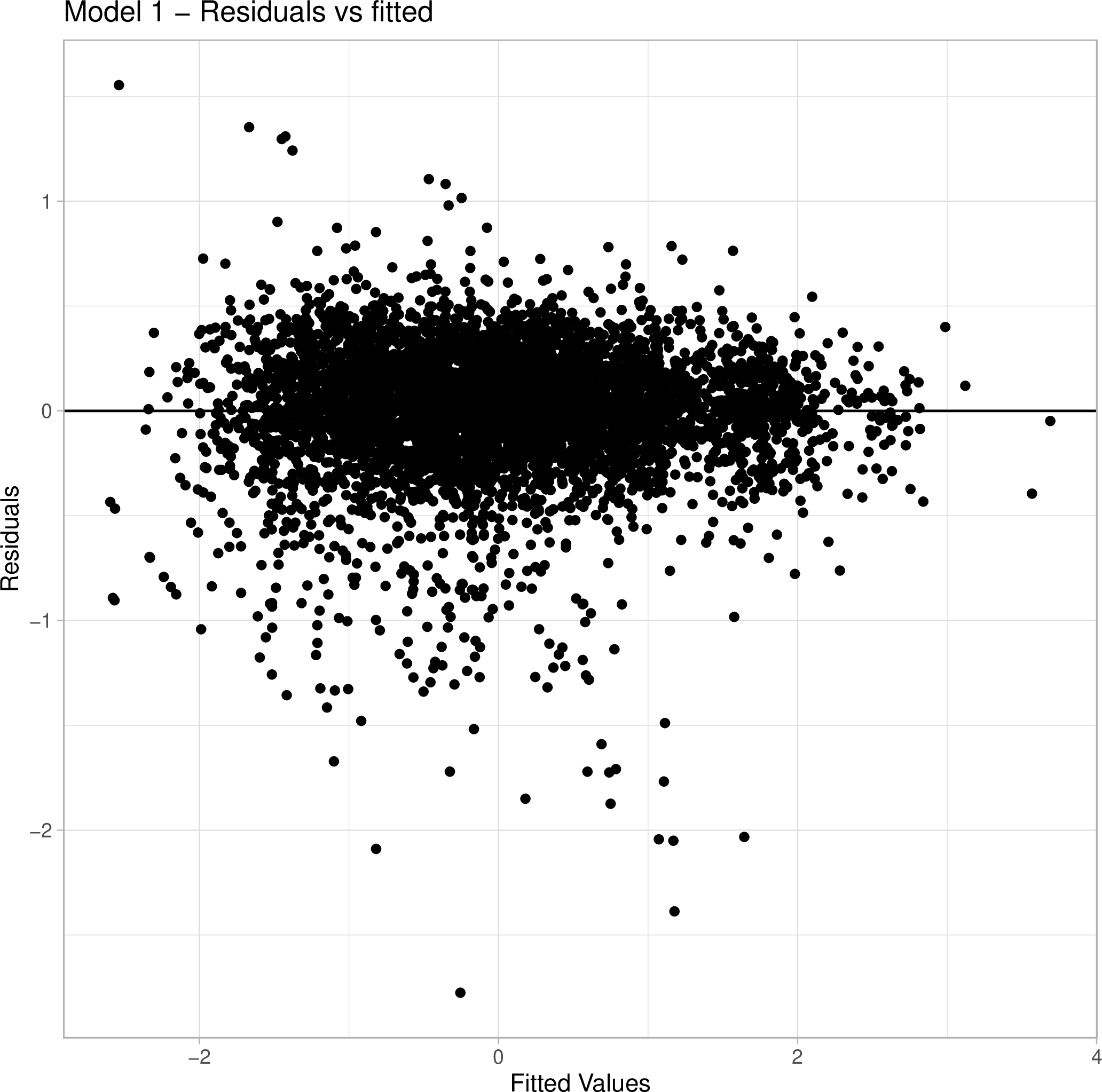

Supplement: Fig S2 — Residuals vs. fitted values plot for Model 1, illustrating the linearity and homoscedasticity of the residuals. (TIFF) [file pone.0323500.s003.tif]

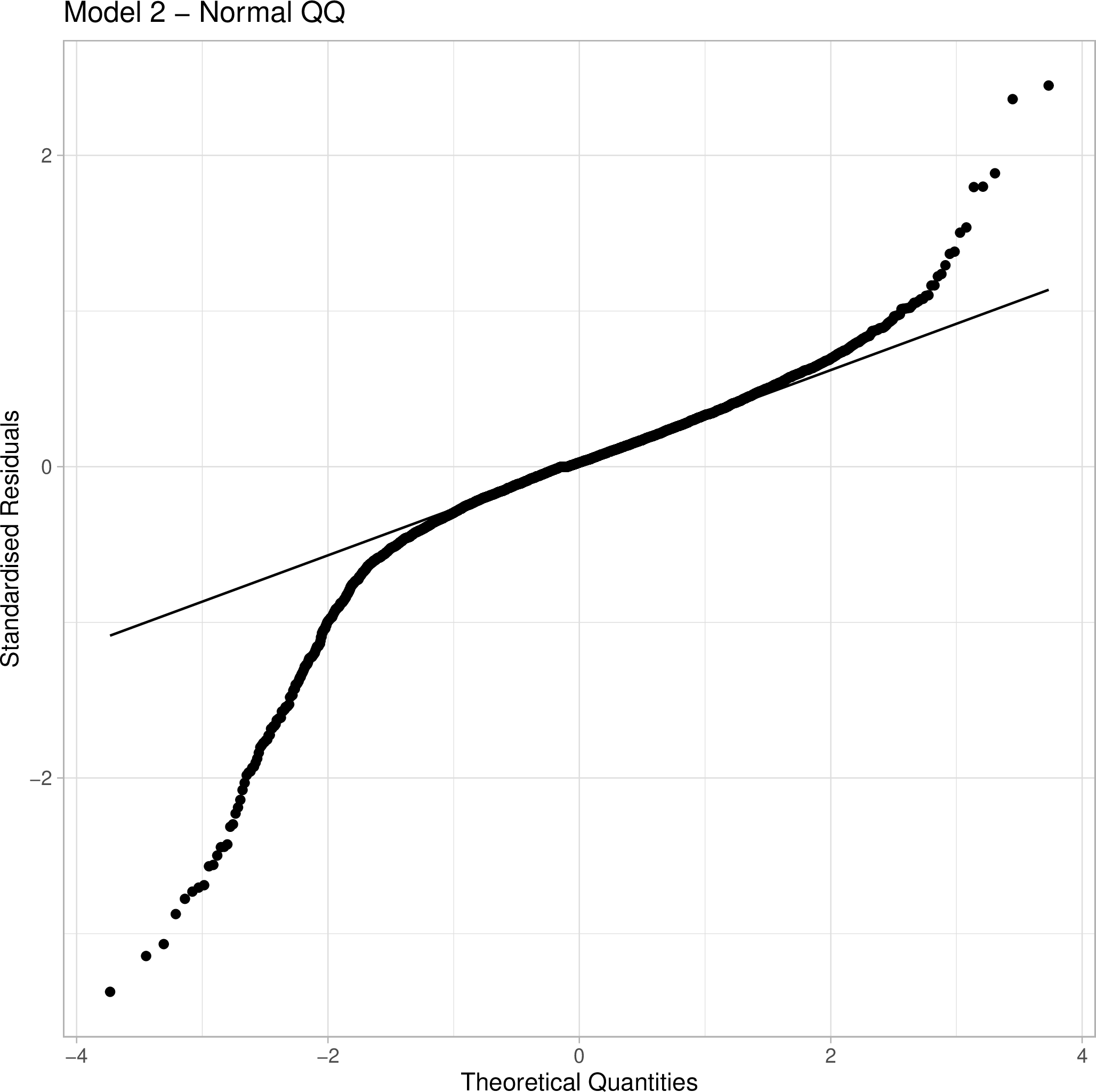

Supplement: Fig S3 — Quantile-Quantile (QQ) plot of Model 2 residuals, showing how closely the residuals align with a normal distribution. Deviations from the line indicate departures from normality. (TIFF) [file pone.0323500.s004.tif]

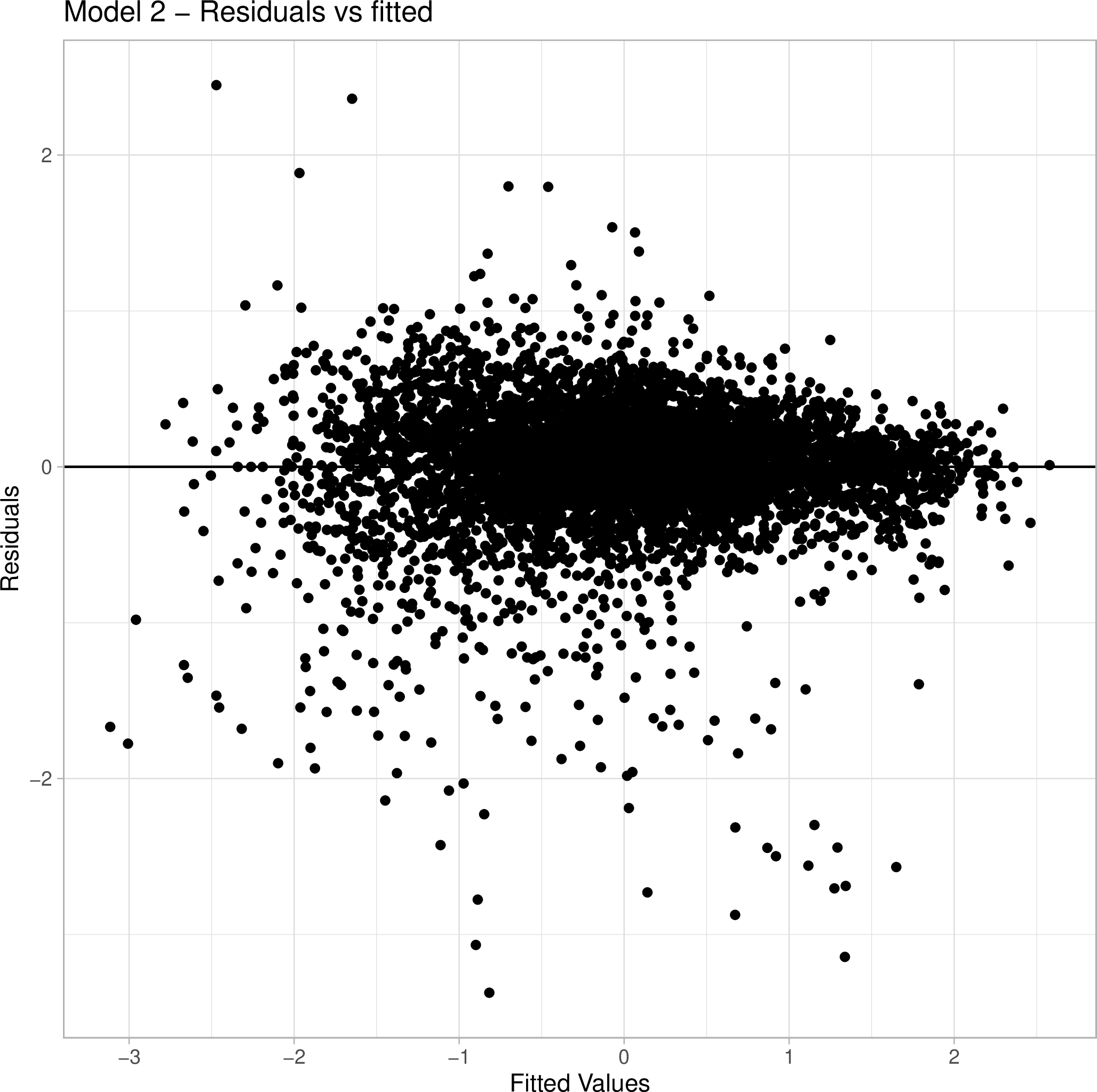

Supplement: Fig S4 — Residuals vs. fitted values plot for Model 2, illustrating the linearity and homoscedasticity of the residuals. (TIFF) [file pone.0323500.s005.tif]

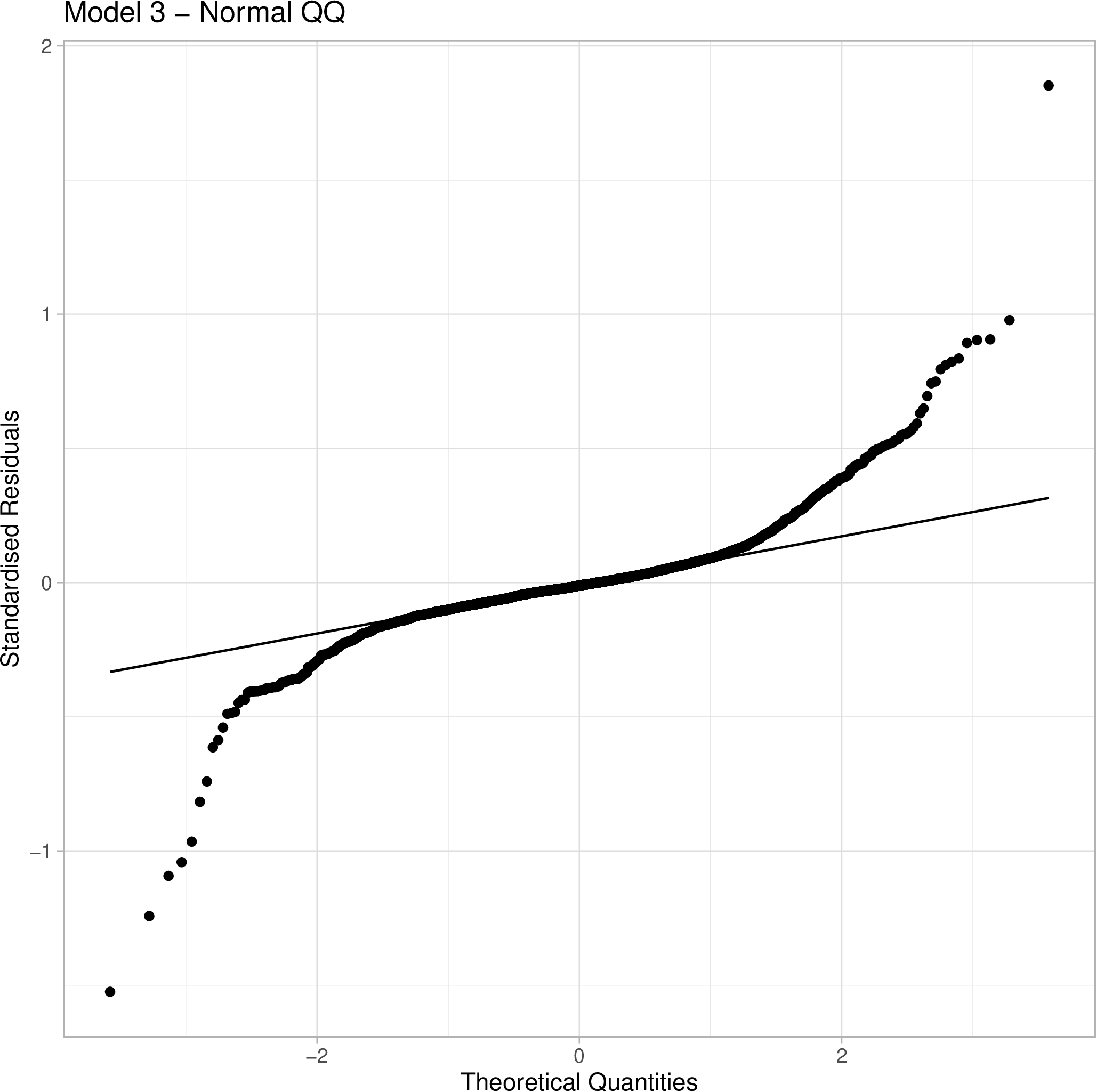

Supplement: Fig S5 — Quantile-Quantile (QQ) plot of Model 3 residuals, showing how closely the residuals align with a normal distribution. Deviations from the line indicate departures from normality. (TIFF) [file pone.0323500.s006.tif]

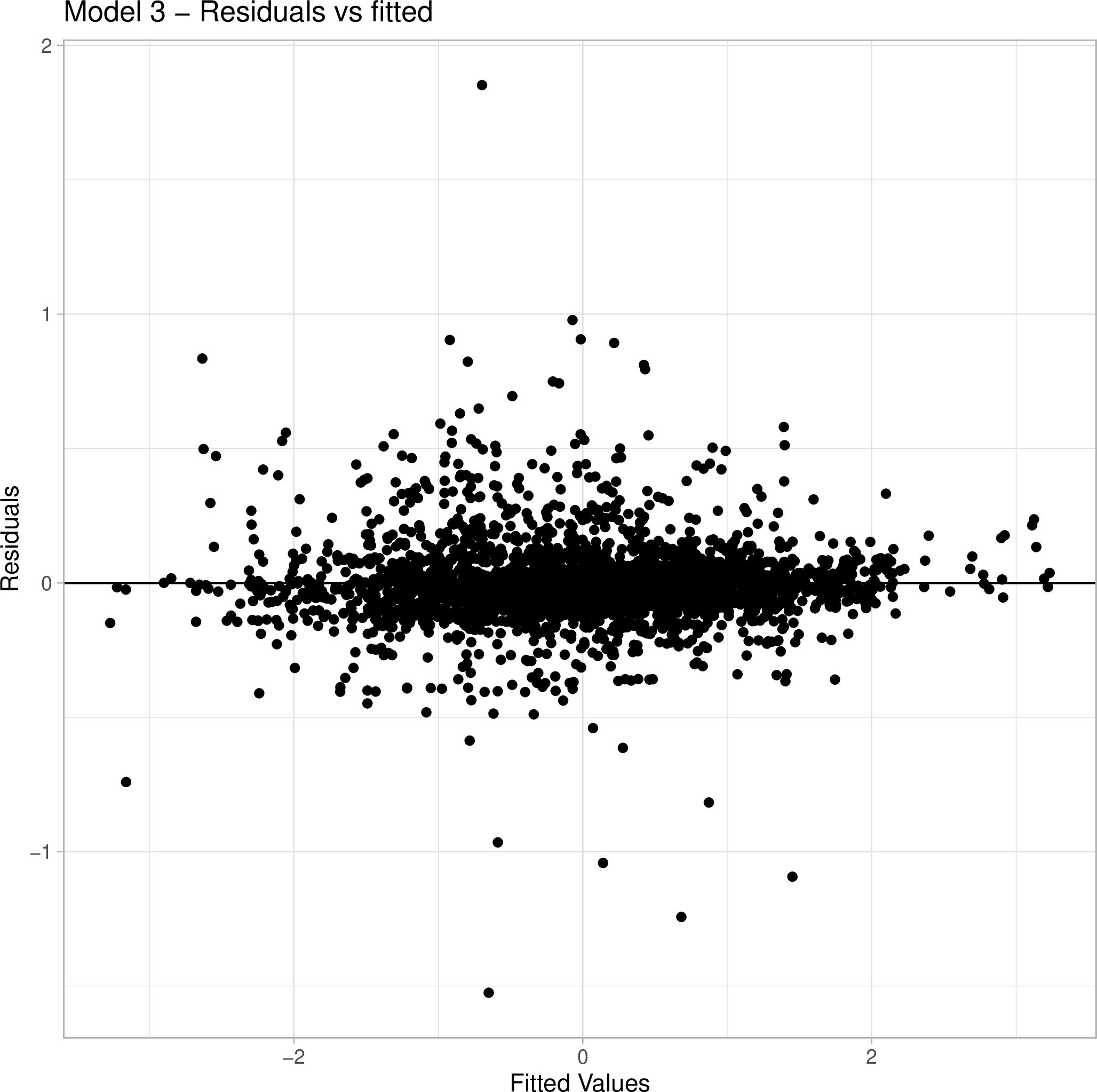

Supplement: Fig S6 — Residuals vs. fitted values plot for Model 3, illustrating the linearity and homoscedasticity of the residuals. (TIFF) [file pone.0323500.s007.tif]

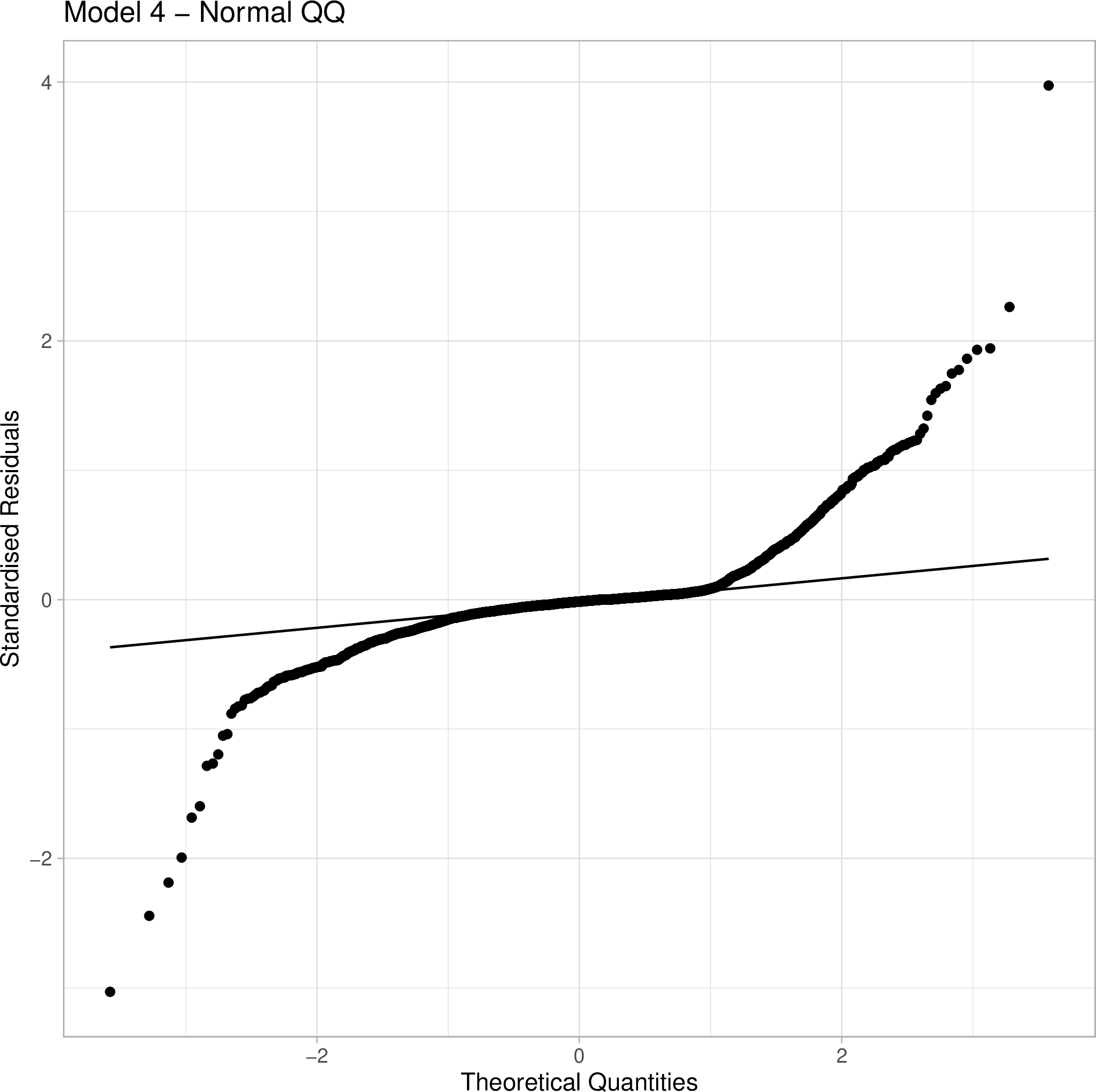

Supplement: Fig S7 — Quantile-Quantile (QQ) plot of Model 4 residuals, showing how closely the residuals align with a normal distribution. Deviations from the line indicate departures from normality. (TIFF) [file pone.0323500.s008.tif]

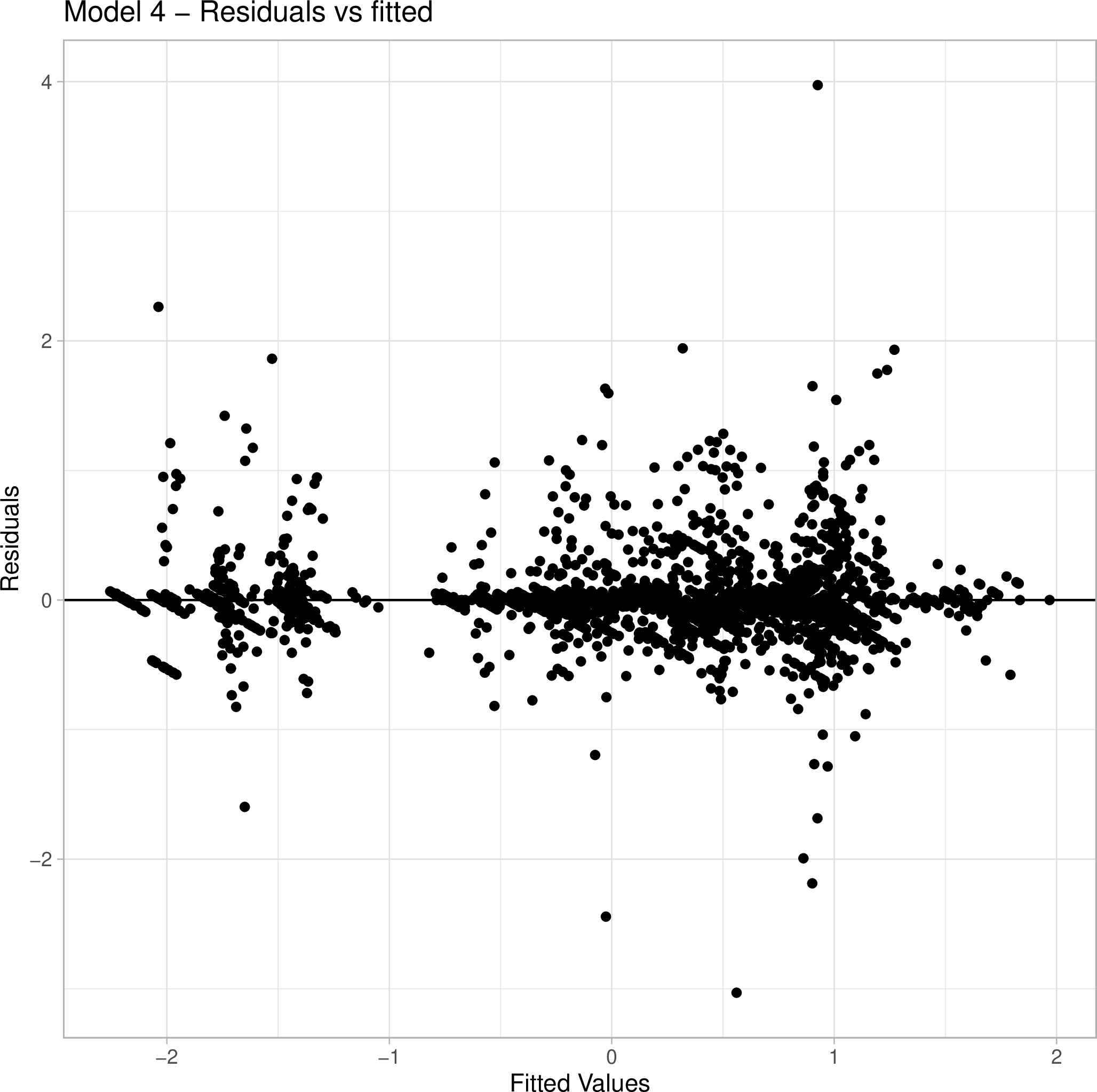

Supplement: Fig S8 — Residuals vs. fitted values plot for Model 4, illustrating the linearity and homoscedasticity of the residuals. (TIFF) [file pone.0323500.s009.tif]

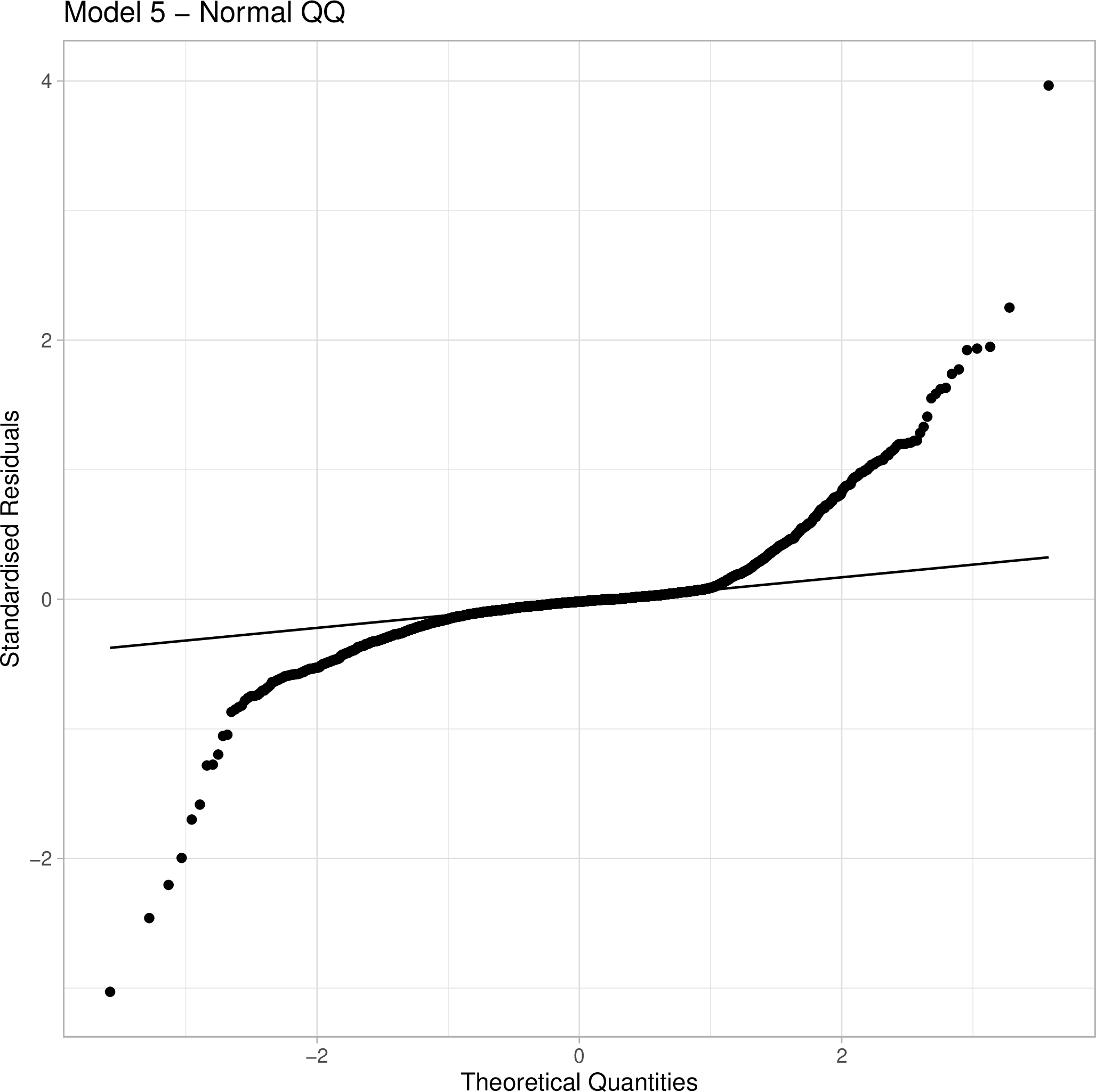

Supplement: Fig S9 — Quantile-Quantile (QQ) plot of Model 5 residuals, showing how closely the residuals align with a normal distribution. Deviations from the line indicate departures from normality. (TIFF) [file pone.0323500.s010.tif]

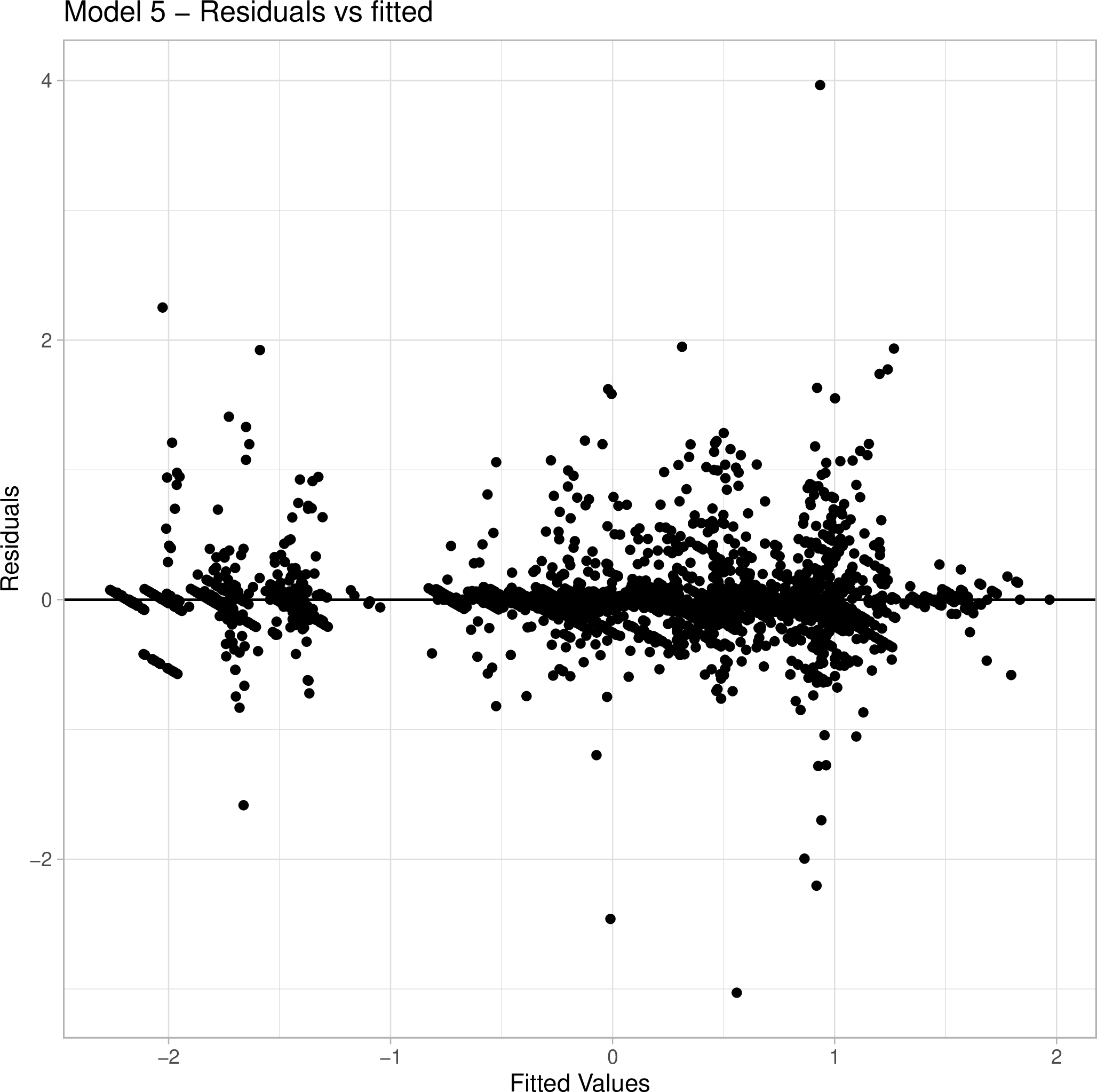

Supplement: Fig S10 — Residuals vs. fitted values plot for Model 5, illustrating the linearity and homoscedasticity of the residuals. (TIFF) [file pone.0323500.s011.tif]
