# Supplementary material for: The influence of resource use on yield versus sale price trade-off in Australian vineyards
Source: PLoS One. 2025 Jun 5;20(6):e0323500. doi: 10.1371/journal.pone.0323500 (PMC12140259; doi:10.1371/journal.pone.0323500)
Supplement: Attachment — Submitted filename: Response to reviewers.pdf [file pone.0323500.s012.pdf]

Response to Reviewers

We are grateful to the reviewers and the editor for their detailed and thoughtful feedback. Each suggestion has helped strengthen the manuscript, and we have made extensive revisions in response to all comments. The updated manuscript is now more clearly connected to vineyard outcomes and climate considerations, benefiting significantly from this review process. We believe these improvements enhance the manuscript’s overall contribution to understanding the trade-offs in resource use and economic productivity within Australian winegrowing regions.

Below, we address each comment individually, outlining the actions taken in response and specifying changes made to the manuscript. All page and line references relate to the revised manuscript (marked-up copy).

Editor’s Comments

We appreciate the editor's guidance and attention to formatting details, which have helped ensure our manuscript aligns with PLOS ONE's style requirements.

When submitting your revision, we need you to address these additional requirements.

1. *Please ensure that your manuscript meets PLOS ONE's style requirements, including those for file naming. The PLOS ONE style templates can be found at:*

Formatting Sample - Main Body

Formatting Sample - Title, Authors, Affiliations

Response:

In compliance with PLOS ONE's style requirements, we have made the following formatting changes:

- Updated all figure references from "Figure" to "Fig" to align with journal standards.
- Arranged tables and graphs to appear immediately after the paragraphs in which they are first mentioned, ensuring smoother readability and compliance with journal style.
- Adjusted the capitalisation in headings to meet the formatting guidelines.
- Added the “S” prefix to supplementary materials in the appendix as suggested by Reviewer 3 and as per PLOS ONE’s requirements.

Thank you for bringing these to our attention.

2. *Please provide additional details regarding participant consent. In the ethics statement in the Methods and online submission information, please ensure that you have specified (1) whether consent was informed and (2) what type you obtained (for instance, written or verbal, and if verbal, how it was documented and witnessed). If your study included minors, state whether you obtained consent from parents or guardians. If the need for consent was waived by the ethics committee, please include this information.*

   If you are reporting a retrospective study of medical records or archived samples, please ensure that you have discussed whether all data were fully anonymized before you accessed them and/or whether the IRB or ethics committee waived the requirement for informed consent. If patients provided informed written consent to have data from their medical records used in research, please include this information.

Response:

Data was not collected as part of this study and was only analysed. Participant consent is managed by the Australian Wine Research Institute (AWRI), which serves as the data custodian. Consent is informed, and participants agree to the terms and conditions provided by Sustainable Winegrowing Australia when signing up as part of the program, which includes data privacy and usage policies. The specific terms and conditions regarding data handling and privacy can be reviewed at the following links:

- Sustainable Winegrowing Australia Terms and Conditions
- Sustainable Winegrowing Australia Privacy Policy

These documents outline the informed consent process and data privacy practices in accordance with AWRI's guidelines. No minors, animals, persons or biohazards were involved in the analysis.

3. *You indicated that ethical approval was not necessary for your study. We understand that the framework for ethical oversight requirements for studies of this type may differ depending on the setting and we would appreciate some further clarification regarding your research. Could you please provide further details on why your study is exempt from*

the need for approval and confirmation from your institutional review board or research ethics committee (e.g., in the form of a letter or email correspondence) that ethics review was not necessary for this study? Please include a copy of the correspondence as an ""Other"" file.

Response:

Thank you for the concern regarding ethics. The study involved no direct interaction with human participants, animals or biohazards and does not present any sensitive or identifiable personal data.

We have reached out to our ethics committee and included further correspondence from them regarding this study's exemption (ethics.eml and ethics.pdf).

4. *Thank you for stating the following financial disclosure: "The corresponding author receives a scholarship stipend as part of their PhD program. This is funded by the Food Agility CRC." Please state what role the funders took in the study. If the funders had no role, please state: "The funders had no role in study design, data collection and analysis, decision to publish, or preparation of the manuscript." If this statement is not correct you must amend it as needed. Please include this amended Role of Funder statement in your cover letter; we will change the online submission form on your behalf.*

Response:

The funders had no role in study design, data collection and analysis, decision to publish, or preparation of the manuscript.

We have included this amended statement regarding the role of the funders in the cover letter, as requested.

5. *Thank you for stating the following in the Competing Interests section:*

   "Mardi Longbottom is employed by Sustainable Winegrowing Australia."

   Please confirm that this does not alter your adherence to all PLOS ONE policies on sharing data and materials, by including the following statement: "This does not alter our adherence to PLOS ONE policies on sharing data and materials." (as detailed online in our

guide for authors http://journals.plos.org/plosone/s/competing-interests). If there are restrictions on sharing of data and/or materials, please state these. Please note that we cannot proceed with consideration of your article until this information has been declared.

Please include your updated Competing Interests statement in your cover letter; we will change the online submission form on your behalf.

Response:

Although not used directly in this study, the data supporting this study contains sensitive financial and identifying information and, therefore, is not publicly available. This part of the terms and conditions mentioned previously is outlined here. Unfortunately, as part of accessing it a non-disclosure agreement is required. However, researchers may request access to the dataset through Sustainable Winegrowing Australia, which manages the data. For access inquiries, please contact Sustainable Winegrowing Australia via the contact page. The corresponding author, Bryce Boyd, is also available to assist in facilitating data access for researchers interested in further analyses. Mardi Longbottom has also extended their support in assisting other researchers access this data with high hopes that further research collaborations can be conducted. These email addresses are persistent to connect with the SWA should they cease being associated with the organisation.

6. *We noted in your submission details that a portion of your manuscript may have been presented or published elsewhere. [Some of the same variables such as water use, vineyard size and yield have been used along with further financial data and other environmental variables as part of a future research piece to model vineyard factor's interactions and how they relate to operating cost and revenue.] Please clarify whether this publication was peer-reviewed and formally published. If this work was previously peer-reviewed and published, in the cover letter please provide the reason that this work does not constitute dual publication and should be included in the current manuscript.*

Response:

No part of this current manuscript has been published or submitted for publication elsewhere. No analyses, text, or research questions overlap with any other work. A different manuscript using some overlapping variables from the same dataset (such as water use, vineyard size, and yield), has been submitted to another journal and is currently

undergoing peer review. The focus of that study is on statistical relationships between vineyard factors and their direct impact on operational costs and revenue, utilising machine learning to model these interactions.

The current manuscript, however, emphasises the environmental and productivity trade-offs in Australian winegrowing regions, integrating considerations of resource use, climate impacts, and sustainability outcomes. Therefore, this manuscript does not constitute dual publication but rather complements the previous research.

We trust this clarifies the distinct focus and contribution of the current manuscript. We are happy to provide a confidential draft of the other manuscript if the editor requires this.

7. *Please include captions for your Supporting Information files at the end of your manuscript, and update any in-text citations to match accordingly. Please see our Supporting Information guidelines for more information: http://journals.plos.org/plosone/s/supporting-information.*

Response:

We updated the captions in the appendix to conform with PLOS ONE's requirements. Each caption now includes the preceding "S" and is renumbered starting from 1. All in-text citations for these files have been updated accordingly.

Reviewers' Comments:

Reviewer 1

1. *Please provide the dataset underlying your publication. Anonymized data should be provided for the factors yield, area harvested, year, GI region (anonymization: regions 1-55), water used, scope one emissions (subset 1) and average sale prices (subset 2).*

As stated in the cover letter and above, the data cannot be shared publicly as it is third-party data that contains sensitive finance and identity information. The authors of this manuscript do not own or have any rights to the data and have signed non-disclosure agreements to handle the data. In line with the third-party data guidelines of PLOS One the data is available from Sustainable Winegrowing Australia, who can be reached through their website contact details here. The corresponding authors Bryce Polley and Mardi Longbottom are willing to help in further connecting researchers to this data to help improve food security and technological advancements that may be gained from further analyses. To support this, Dr Longbottom has also added their email to the contact list.

The authors would also like to note that prior researchers have been successful in accessing this dataset when liaising with Sustainable Winegrowing Australia. And further partnerships have begun to develop between other researchers and institutions beyond QUT to do further analyses on the data that Sustainable Winegrowing Australia have collected.

2. *Lines 63-64: please provide more information about how and in which occasion winegrowers provided their data in the web interface.*

We have expanded the Methods section to include further details on how and when winegrowers entered their data into the web interface. Data was recorded manually by winegrowers via a web-based interface provided by Sustainable Winegrowing Australia (SWA). We also referenced the SWA user manual, which outlines the data entry process and interface.

New text (Line 78): Data recorded by Sustainable Winegrowing Australia were entered manually by winegrowers voluntarily using a web-based interface. Data was recorded after the Australian crush (February to April), with entries due for the season by the 31st of August each year. The SWA user manual outlines details surrounding the web interface and its requirements [32]. Vineyards volunteered data as part of Sustainable Winegrowing Australia initiatives, which included collaboration, workshops and certification of

sustainable practices using third-party auditors. Vineyards were only included for each model if they recorded all the variables used for the corresponding model (see Table 1). Each vineyard had at least recorded region, harvest year, yield and area harvested. Other variables used but not present for every vineyard were average sale price, water used, and fuel used (diesel, petrol, biodiesel and LPG). Fuel use was converted to equivalent tonnes of carbon dioxide and collectively referenced as emissions to enable direct comparisons between fuels. All variables were continuous except for harvest year and region, which were categorical variables (Table 1).

3. *Lines 73-74: Please provide reference or provide regional average prices, which were used to complete the dataset.*

We have clarified in the manuscript that regional average prices used to complete the dataset were sourced from Wine Australia's publicly available data. We have included references to the relevant Wine Australia Annual Reports to support this data source.

<u>New text (Line 94)</u>: As data from Sustainable Wine Australia were voluntarily given, missing values were improved using regional average prices from the Wine Australia (previously the Winemakers Federation of Australia) data. Data from Wine Australia were collected via phone surveys and included total tonnes purchased, the average cost per tonne, and yearly change in price for region and grape varietal; the data is publicly available through the Wine Australia Annual reports [19, 20, 33–41].

4. *Lines 123-124: Which limits were set in order to group the different regions into cool, hot, mild, warm, or damp, dry, very dry regions? Please add the information here.*

We have added a description of the classification criteria and described the process undertaken for their derivation. We further add the reference to the work where this was done Coombe & Dry 2010. We also further guide readers to the Wine Australia climate atlas that documents many supplement measures for every GI Region.

<u>New text (Line 149):</u> Each GI Region's climatic properties were summarised using predefined classifications per the [32] user manual. The user manual describes climates by rainfall and temperature, creating supersets of regions of similar climatic properties. The climatic groups illustrated similarities and differences in sets larger than GI Regions.

These classifications were literal descriptors used by industry members to discern between different weather types within winegrowing regions. However, the climate descriptors for Australian GI regions were originally conceived by [49] using temperature-based indices. A key metric employed was the Mean January Temperature (MJT), which

indicates heat accumulation during the growing season. Regions were categorised based on their MJT values into cool, intermediate, warm, and hot climates. This classification was used to aid viticulturists in selecting grape varieties best suited to the thermal conditions of each region. Further in-depth summarisations of Australian wine region climates can be found in greater detail using Wine Australia's climate atlas [50]. Other climatic descriptors were explored, such as the Köppen climate classification; however, this index did not offer enough granularity to highlight any trends or clustering within the data."

5. *Line 190: Please correct p=0.0.39.*

Thank you for pointing this out. We have corrected the p-value as requested.

New text (Line 254): The three exceptions were scope one emissions in Model 3 (p=0.22) and Model 4 (p=0.39) and the interaction between the area harvested and water used in Model 2 (p=0.22)

6. *Fig. 4: Please convert into absolute numbers (yield [t ha $^{-1}$]; average price [AUD ha $^{-1}$]) to make results more transparent.*

We have updated the axes on Figure 4 to display absolute values to improve clarity and transparency in presenting the results.

7. *Line 240: Please add that the strong negative correlation between vineyard size and average sale price could be due to regionally differing vineyard sizes – therefore please cancel lines 356-358.*

Thank you for this insight. We have added an explanation and have additionally removed lines from the conclusion as requested. This change also aligned well with edit 19 to better refine the conclusion.

New text (Line 315): Additionally, the strong negative correlation between vineyard size and average sale price could be due to regionally differing vineyard sizes, with warmer inland regions tending to have much larger vineyard sizes.

8. *Line 248: Please comment that scope one emissions are only responsible for a small portion of energy input in vineyards and that other inputs significantly contribute to the carbon footprint of grape production. Please add further references (e.g., Rugani et al., 2013).*

Thank you for this suggestion. We have clarified that scope one emissions contribute only a small portion of the total energy input in vineyards, with other inputs, such as fertilisers,

pesticides, and electricity, playing a significant role in the carbon footprint of grape production. Additionally, we have referenced Rugani et al. (2013) to emphasise the complexity and uncertainty of interpreting emissions across viticulture practices, as vineyard size and operational practices can substantially impact carbon dynamics.

New text (Line 362): It is difficult to directly discern the connections between scope one emissions, as fuel can be used for a broad category of activities. As 69 highlights, the interpretation of emissions is uncertain and inexplicit when comparing viticulture practices. Many factors, especially size, can directly or indirectly influence the dynamics of carbon emissions in a vineyard's life cycle. Notably, scope one emissions account for only a small portion of the total energy input in vineyards, as other significant inputs, such as fertilisers, pesticides, and electricity, collectively contribute to a substantial share of the carbon footprint in grape [70].

9. *Please comment on the relation between temperature and yields in the discussion and add respective references for it.*

Thank you for this suggestion. We have added a new paragraph discussing the relationship between temperature and yield. This paragraph explains how climate, particularly temperature, affects yield and wine quality. For example, in warmer regions, the ripening period may shorten, and harvests during high-temperature periods can negatively impact both yield and wine quality.

New text (Line 319: The difference between regions can also be due to varied reasons, such as climate; where warmer regions result in a shortening of the ripening period and the risk that if harvest occurs in a duration of high temperature, there could be a negative impact on wine quality [57–59] and yield [60, 61]. Climate change might move the north and south latitude boundaries of areas suitable for good quality wines [62]. It could even lead to improvements in fruit production and quality improvements in some areas [63]. However, other areas may be negatively affected by high temperatures and water stress due to reduced water availability. There is significant room to investigate the impact of different climate scenarios on these other regions and further the various consequences of climate change, specifically for water in the Australian context.

10. *Please insert a Figure caption for figure "yield versus value." Are yields used here scaled to area? Please use absolute numbers [t ha $^{-1}$]. Why did you choose to*

correlate yield average price to it? Please use absolute numbers for depicting the correlation in order to make your findings more transparent.

Thank you for your comments on this figure. The “yield versus value” figure was initially created for internal comparison during our analysis, and we considered including it as supplementary material. However, based on your feedback and the need for clarity, we decided to remove it, as it did not add directly to the main findings of the manuscript. We appreciate your input, which helped us make this decision to focus on the most relevant data.

Reviewer 2:

11. *The paper analyzes a database of 10 years and 1,261 Australian vineyards. The authors seek to establish a relationship between resource use, yield, and sale price. The authors use five general linear models to capture the relationship of resources related to water and fuel use with the output yield and average sale price of the resultant product, taking into account the size and location of the vineyard. The R^2 values of the models are, in general, quite high. However, there are so many aspects of the problem that it is difficult to arrive at very useful conclusions, which would need to be more specific.*

Thank you for your acknowledgement of the challenge in deriving specific conclusions given the complexity of vineyard operations across different contexts. In our revisions, we have incorporated some of these considerations by highlighting targeted techniques, such as mulching and undervine practices, that align with the climatic constraints and operational focuses of various regions. We incorporated these alterations within other edits such as the addition of talking about climatic clusters and additions to the discussion (see edits 12, 18 and 9).

12. *It seems that it might be better to try to divide the vineyards into clusters that share some similarities, such as size, geographical indicators, etc. This might lead to more useful conclusions for the vineyard managers, like certain strategies that could be recommended for vineyards of a certain cluster.*

Thank you for this helpful suggestion. We have added a figure (Fig. 5, line 287) that illustrates clusters of vineyards, specifically distinguishing cooler, damper coastal regions from warmer, drier inland regions. Additionally, we included a paragraph discussing how these clusters reflect distinct coastal and inland trends, which could benefit from tailored management strategies.

New text (Line 329): The findings from this study reveal notable differences in yield and profitability between vineyard clusters based on climate conditions, specifically between the warm/hot regions and cooler/damper regions, with warmer drier regions tending to be further inland in Australia, compared to the more coastal cooler and damper regions. Warmer areas are characterised by longer growing seasons and higher heat accumulation, and are generally associated with higher yields per hectare (as shown by the results in Fig 4 and 5. However, these conditions often entail trade-offs in grape quality, as accelerated ripening due to higher temperatures can reduce the complexity of flavours—a key determinant of premium wine quality [64, 65]. Consequently, grapes from warmer regions tend to be lower-priced, with an emphasis on yield rather than [66]. By contrast, cooler coastal regions, which benefit from moderate temperatures and oceanic cooling, produce lower yields per hectare but command higher prices due to the slower ripening process that fosters more nuanced flavours in the grapes [60, 65].

13. *The authors should also check for typos. I found "hoiwever" instead of "however."*

Thank you for pointing this out. We ran a spelling and grammar check over the entire manuscript and conducted a manual review to ensure accuracy.

14. *In this paper, the authors analyze the relationship between resource use, yield, and sales price using statistical models. Specifically, they unveil an interesting interplay between size, resource allocation, climate, and regional influences in shaping sales price and yield.*

Thank you for your insightful engagement with our work. We appreciate the careful attention you've given to the central themes of our analysis.

15. *In the introduction section, establish the contributions of this paper.*

Thank you for this suggestion. We have added a paragraph in the introduction to clarify the contributions of our study.

New text (Line 54): We aim to contribute to understanding resource-use efficiency in the Australian wine industry by providing and analysing these baselines using this unique set of regional and temporal data on vineyard resource allocation, grape yield, and sales price. Our analyses reveal that resource input per area, rather than overall expenditure, is a more

accurate predictor of high sales prices, especially in regions with specific climate and size characteristics. We further aim to link this effect to regional attributes, where climatic factors interact with operational scale. These contributions will help to establish a baseline for vineyard managers and policymakers to assess resource trade-offs, ultimately supporting more sustainable and economically viable decision-making in the face of climate and market pressures.

16. *In the methods section, justify the choice of methods employed. A framework chart specifying inputs and output may be helpful to include.*

Thank you for this suggestion. We have added an extra paragraph in the Methods section to highlight our justification for the chosen methods with further citations.

<u>New text (Line 168):</u> General Linear Models (GLMs) were chosen as the primary analytical approach due to their ability to quantify and interpret relationships between continuous predictor variables (such as water use and emissions) and the continuous response variables of interest (yield, yield per hectare, and average sale price). GLMs offer the flexibility to examine both main effects and interaction terms [51], which is essential for exploring the nuanced interplay between resource use, vineyard size, and regional factors. Many prior studies have used GLMs to model vineyard relationships for response variables such as yield [52, 53]. Additionally, GLMs allow us to incorporate categorical variables (Year and GI Region) to account for temporal and spatial heterogeneity, making it possible to observe differences in vineyard performance across diverse Australian winegrowing regions over time. The interpretability of GLM coefficients makes this approach attractive for practical applications in vineyard management [54], providing insights into the magnitude and direction of each variable's influence on yield and sale price outcomes (given the other variables in the model).

17. *In the results section, provide the p-values for all coefficients in Table 5 and reference properly the figures and tables provided in the Appendix.*

We have added the p-values for all coefficients in Table 5 as requested. Additionally, we have updated the numbering convention and corrected references for figures and tables in the Appendix to ensure clarity and consistency as the editor has also requested.

18. *In the discussion section, provide some practical implications of this study for winegrowers and wine supply chain management.*

Thank you for this valuable suggestion. We have added a new paragraph in the Discussion section to outline practical implications of our findings. This includes considerations on

the viability of scaled grape production alongside sustainable strategies, highlighting how resource efficiency and regional climate factors can guide decision-making for winegrowers and supply chain managers.

<u>New text (Line 439):</u> For winegrowers, understanding the inverse relationship between vineyard size and average sale price suggests that smaller vineyards may benefit from focusing on high-value, lower-yield practices, which allow for a more targeted approach to quality management. In larger vineyards, where economies of scale may drive profitability, strategies that optimise resource use efficiency per hectare become crucial, especially in regions facing water scarcity. Here, larger vineyards could benefit from adopting further sustainable and climate adaptive practices such as mulching [67], vineyard canopy and mid-row management strategies [67], and soil nutrient leaching and organic management strategies [71, 81, 82] to help aid in water, fuel and fertiliser reductions. Given the climatic pressures in warmer inland regions, especially those with high water requirements, these practices may also mitigate the risk of crop failure due to drought or heat stress, enhancing both resilience and economic viability. Additionally, international market trends increasingly favour sustainably sourced wine, suggesting that supporting vineyards in sustainable water and fuel management could enhance export opportunities, meeting both environmental goals and market expectations.

19. *Finally, in the conclusion section, provide some promising subjects for further investigation.*

Thank you for this suggestion. We have expanded the conclusion to include a segment on future research directions. We also found this aligned well with edit 7 in refining the conclusion.

<u>New text (Line 490):</u> This study delved into the relationships between resource use, grape sales price and yield. The findings underscore the multifaceted nature of vineyard management, where the interplay of size, resource allocation, climate, and regional influences collectively shape both the expected sale price and the quantity of grape yields. The average sales price of grapes was not solely tied to the overall expenditure of resources but rather to the efficient allocation of resources per area. This emphasises that factors beyond sheer scale contribute significantly to the final sale price of grapes produced. Moreover, regional and yearly variations substantially affected vineyard outputs, impacting sales price and quantity. Building on these insights, future research could link these types of interpretable models to other studies using machine learning techniques and causal inference to improve accuracy and link sustainable practice would

greatly strive toward the creation of decision support tools to further inform winegrowers of sustainable options and their efficacy.
